# Novel Chiral Self-Assembled Nano-Fluorescence Materials with AIE Characteristics for Specific Enantioselective Recognition of L-Lysine

**DOI:** 10.3390/ijms251910666

**Published:** 2024-10-03

**Authors:** Peng Wang, Rong Wang, Yue Sun, Yu Hu, Kaiyue Song, Xiaoxia Sun

**Affiliations:** 1 Jiangxi Province Key Laboratory of Organic Functional Molecules, Institute of Organic Chemistry, Jiangxi Science and Technology Normal University, Nanchang 330013, China; 13970096103@163.com (P.W.); wr19991202@163.com (R.W.); 2 State Key Laboratory of Molecular Engineering of Polymers, Shanghai Key Laboratory of Molecular Catalysis and Innovative Materials iChEM, Department of Chemistry, Fudan University, Shanghai 200433, China; sunyue19980307@163.com; 3 College of Chemistry and Chemical Engineering, Nanchang University, Nanchang 330031, China; huyu@ncu.edu.cn

**Keywords:** self-assembled, chiral nano-fluorescence, aggregation-induced emission (AIE), enantioselective recognition, lysine

## Abstract

In this paper, two aggregation-induced emission (AIE) chiral fluorescent materials, S-1 and S-2, were synthesized. The two materials are based on BINOL and H_8_-BINOL backbones, respectively, and large electron-absorbing groups are attached to the chiral backbones through the Knoevenagel reaction. At the same time, the CD signals of these two chiral fluorescent materials are gradually weakened (f_w_ gradually increases) as they continue to aggregate. However, S-2 underwent a flip-flop from a negative to positive chiral CD signal at f_w_ ≥ 90. And both materials also showed significant enantioselective recognition of lysine, demonstrating their potential as novel chiral fluorescent probes. Among them, the enantioselective fluorescence enhancement ratios (ef) of S-1 and S-2 for lysine were 10.0 and 10.3, respectively, while different degrees of blue shifts were produced by the ICT mechanism during the recognition process. In addition, the self-assembled morphology of the two nanomaterials is different; S-1 comprises hollow-core vesicles that are more likely to aggregate to form larger self-assembled vesicles, whereas S-2 is a solid block structure. When L/D-lysine was added alone, the morphology of S-1 was more distinctly different compared to S-2. With the addition of L-lysine, S-1 was dispersed and regularly spherical, whereas with the addition of D-lysine, S-1 itself remained in the form of aggregated large vesicles. This suggests that both S-1 and S-2 are important in the fields of chiral optics, chiral recognition, and nanoscale self-assembly.

## 1. Introduction

Chiral nanomaterials exhibit unique characteristics that make them particularly suitable for applications in biosensing and catalysis [1,2,3]. One of the key properties of chiral nanomaterials that make them particularly effective in these applications is their ability to bind to chiral molecules with high affinity. This helps in enhancing the sensitivity and detection limit of biosensors, making them capable of detecting even minute amounts of target analytes [4,5]. In addition, chiral nanomaterials exhibit chirality-induced optical activity, which can be used to detect and quantify target analytes. This property allows for the development of highly sensitive and selective biosensors, making them ideal for applications in medical diagnostics and environmental monitoring [6,7,8,9]. Overall, the unique properties of chiral nanomaterials, such as their ability to bind to chiral molecules, high surface area-to-volume ratio, chirality-induced optical activity, and efficient catalysis, make them particularly suited for applications in biosensing and catalysis [10,11,12].

Fluorescent organic small-molecule materials are employed in a multitude of applications, including the fabrication of optoelectronic components, the design of organic fluorescent probes, environmental sensors, information encryption, and a plethora of other fields [13,14,15,16]. In the context of traditional fluorophores, it has been observed that their fluorescence in solution is robust, yet once aggregation or the solidification of the fluorophores occurs, the fluorescence is significantly weakened or even extinguished, a phenomenon known as aggregation-causing quenching (ACQ) [17,18,19]. This defect precludes their applicability in luminescence-based applications involving aggregated or thin-film states [20,21]. In 2001, Tang et al. discovered the phenomenon of aggregation-induced emission (AIE), whereby some propeller-shaped molecules appeared to be weakly emitted or non-emitted when dissolved in solution, but became strongly emitted when the molecules were aggregated [22]. Since then, the AIE phenomenon has broadened the application of organic fluorescent materials in organic light-emitting diodes, sensors, cell staining, drug delivery, and other fields [23,24,25,26].

The synthesis of two novel aggregation-induced emission (AIE) chiral fluorescence materials, namely S-1 and S-2, had been successfully achieved via the Knoevenagel reaction involving the commercially available, rigid and well-established chiral BINOL skeleton and H_8_-BINOL building blocks, as well as large electron-withdrawing groups. Significant electron-withdrawing groups played a crucial role in the chiral framework of aggregation-induced emission luminogens by enhancing the electron affinity of the molecules and reducing their HOMO-LUMO gap to 3.50 eV and 3.75 eV, respectively. S-1 and S-2 displayed weak emission in dilute solutions and enhanced emission in the polymerization state, which is indicative of the specific AIE phenomenon. The chiral CD signals of both compounds weakened with the degree of continuous aggregation. Moreover, S-2 underwent a chiral flip-flop when highly aggregated. In addition, both compounds were shown to be enantioselective recognizers of L/D-Lys with enantioselective fluorescence enhancement ratios of 10.0 and 10.3 (10 eq) for S-1 and S-2, respectively. The addition of L-lysine and D-lysine to the probes resulted in changes in their micromorphology and their detailed structures.

## 2. Results and Discussion

### 2.1. Synthesis and Characterization

**Scheme 1:**
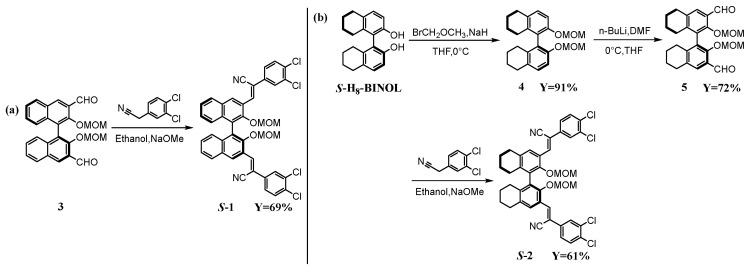
The synthesis steps of compounds (**a**) S-1 and (**b**) S-2.

Commercial chiral S-BINOL and S-H_8_-BINOL are ideal chiral dopants for the synthesis of chiral organic materials. The target compounds S-1 and S-2 were synthesized through the classical Knoevenagel reaction of corresponding BINOL-based aldehydes and 3,4-dichlorophenyl acetonitrile in ethanol under the catalysis of sodium methoxide (Scheme 1). The results consistent with the structure were confirmed by ^1^H NMR, ^13^C NMR, infrared spectrometry, and high-resolution mass spectrometry (Supplementary Materials). The compounds were soluble in common solvents, such as tetrahydrofuran, methanol, and dichloromethane, but insoluble in water.

### 2.2. Self-Identification

**Figure 1:**
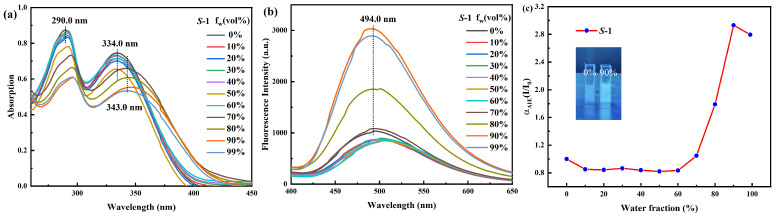
(**a**) UV-Vis absorption spectra of S-1 in THF/H_2_O mixtures (20 μM) with water fractions (0–99%); (**b**) fluorescence emission spectra of S-1 in THF/H_2_O mixtures (20 μM, λ_ex_ = 334 nm, slit: 2.5/5.0 nm) with water fractions (0–99%); (**c**) dependence of I/I_0_ ratios on solvent composition (insert: photograph of S-1 in THF/water mixtures with f_w_ values of 0 and 90% under irradiation with 365 nm UV light).

The photophysical properties of S-1 and S-2 were studied by UV-Vis absorption and fluorescence emission spectra. The UV-Vis spectrum of S-1 in THF solution exhibits the characteristic absorption band of BINOL at 290 nm (Figure 1a), and the absorption band at 334 nm corresponds to the intramolecular charge transfer (ICT) from the electron donor BINOL to the electron acceptor (1-cyano-1-(3,4-dichlorophenyl)), the methylene unit. Upon excitation by light, the diluent S-1 emits a weak emission. Since water is a poor solvent, the absorption and emission behavior of S-1 in a mixture of THF and water in different proportions has been studied. As shown in Figure 1a, with the increase in the water volume fraction (f_w_), the absorption peak at 334 nm had a redshift of ~9 nm, and the absorbance decreased to a certain extent. With the increase in f_w_ in the THF solution, the emission intensity showed a downward trend (Figure 1b) when f_w_ was less than 60%, which may be due to the ICT effect. With the addition of water, the emission intensity was enhanced and the emission peak showed a slight blue shift at 494 nm. The fluorescence intensity increased sharply and reached a peak value when f_w_ reached 90%, 2.9 times higher than that in the pure THF solvent, emitting blue fluorescence (Figure 1c). And the fluorescence quantum yields of S-1 were 2.1% and 7.9% in pure THF and 99% water, respectively.

**Figure 2:**
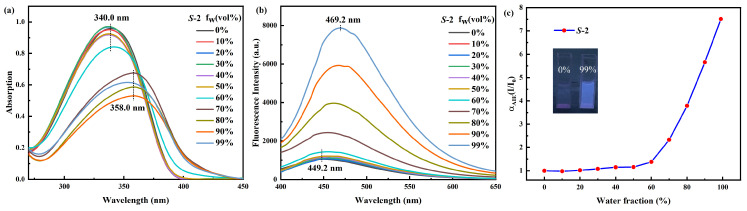
(**a**) UV-Vis absorption spectra of S-2 in THF/H_2_O mixtures (20 μM) with water fractions (0–99%); (**b**) fluorescence emission spectra of S-2 in THF/H_2_O mixtures (20 μM, λ_ex_ = 340 nm, slit: 5.0/5.0 nm) with water fractions (0–99%); (**c**) dependence of I/I_0_ ratios on solvent composition (insert: photograph of S-2 in THF/water mixtures with fw values of 0 and 99% under irradiation with 365 nm UV light).

The H_8_-BINOL-based compound S-2 was designed and synthesized to understand the relationship between molecular structure and AIE properties. Since water is also a poor solvent for S-2, the absorption and emission behaviors of THF and water mixtures in different proportions were studied under the same method. As shown in Figure 2a, the UV-Vis absorption spectrum of S-2 in the THF solution had an absorption peak at 340 nm, and the absorption peak had a redshift of ~18 nm when f_w_ was greater than 60%. When f_w_ was less than 60%, the emission intensity increased slowly. With the increase in water content, the emission intensity increased rapidly and the emission peak had a redshift of ~20 nm at 469.2 nm. When f_w_ reached 99%, the fluorescence intensity reached its peak, which was about 7.5 times higher than that in the pure THF solvent, emitting bright blue fluorescence. Obviously, both S-1 and S-2 are typical AIE luminaires. And the fluorescence quantum yields of S-2 were 1.9% and 11.1% for pure THF and 99% water content, respectively.

**Figure 3:**
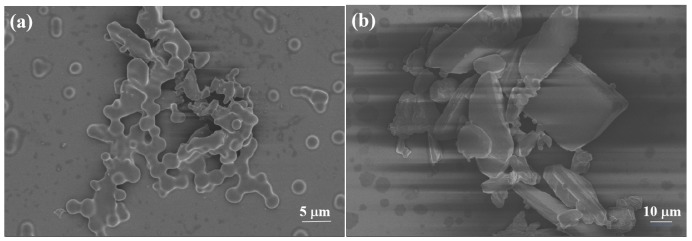

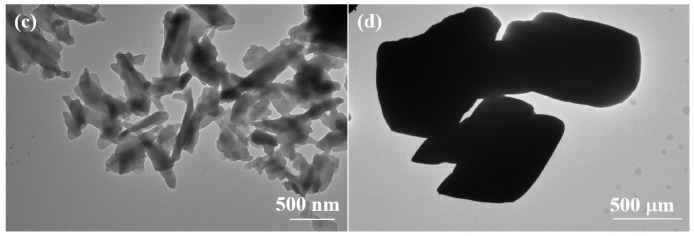
(**a**) The SEM image of S-1 in EtOH. (**b**) The SEM image of S-2 in EtOH. (**c**) The TEM image of S-1in EtOH. (**d**) The TEM image of S-2 in EtOH.

In order to further observe the micro- and nanostructures of S-1 and S-2, we performed SEM and TEM observations. As shown in Figure 3a,c, compound S-1 dissolved in ethanol will obviously undergo a self-assembly effect, forming unique hollow vesicle structures. Moreover, multiple vesicle molecules will autonomously aggregate and stack layer by layer to form a larger irregular vesicle structure. As shown in Figure 3b,d, S-2 self-assembles in ethanol as a distinct stone-like solid. This indicates that S-1 and S-2 self-assemble in the solvent to form different morphologies.

### 2.3. Aggregation-Induced Annihilation of CD Signals

**Figure 4:**
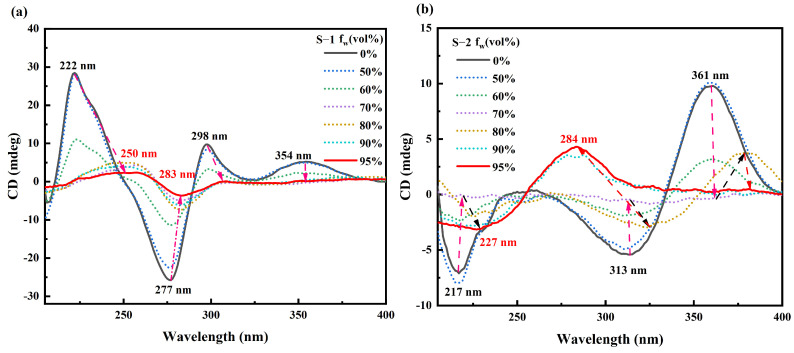
CD spectra of (**a**) S-1 and (**b**) S-2 (100 μM) in THF–water mixtures with different f_w_ values.

It is well known that BINOL and H_8_-BINOL have obvious chiral characteristics. It is also known that S-1 and S-2 have AIE characteristics. Therefore, we used the CD signals of circular dichroism to explore the optical significance of the chirality of the two when they are dispersed and aggregated in the THF-H_2_O mixed system. As shown in Figure 4a, S-1 in the dispersed state (f_w_ = 0%) has significant positive CD signals at 222 nm, 298 nm, and 354 nm, and a significant negative CD signal at 277 nm. The absorption at 354 nm may be due to the transfer of the chiral BINOL unit to the electron-drawing group, while the other absorption peaks are the characteristic absorption peaks of the chiral BINOL group. When fw gradually increases, it can be clearly observed that the intensity of the CD signals at all four sites decreases. When f_w_ = 95%, only the original 277 nm peak still has a CD signal; it is red-shifted to 283 nm, and the rest of the peaks almost disappear.

As shown in Figure 4b, S-2 shows three strong absorption peaks. There are negative signals at 217 nm and 313 nm, and a positive CD signal at 361 nm. Similarly, as fw gradually increases, the CD signal of S-2 gradually decreases and almost disappears. However, after f_w_ ≥ 90%, a clear positive CD signal is generated at 284 nm. We venture to guess that this is due to a chiral flip of the negative CD signal at 313 nm and a blue shift to 284 nm. In conclusion, this result suggests that the chiral CD signals of both S-1 and S-2 diminish as they move from dispersed to aggregated states. In particular, S-2 also undergoes a significant chiral flip at f_w_ ≥ 90%. This indicates that both of them have some significance and application in chiral optical materials. The absolute chiral CD spectra and CPL spectra of the compounds can be seen in the Supplementary Figures S15 and S16.

### 2.4. Recognition Applications

**Figure 5:**
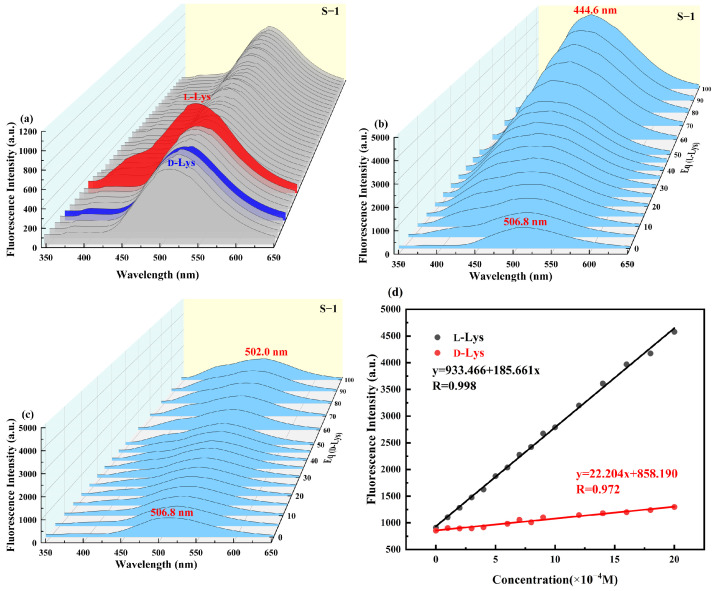
(**a**) Fluorescence emission spectra of S-1 (20 μM) with common amino acids (5 eq) in DMSO solvent; fluorescence spectra of S-1 (20 μM in DMSO, 1.0 eq) with (**b**) L-lysine or (**c**) D-lysine (in H_2_O, 5–100 eq); (**d**) fluorescence intensity of S-1 changed with increase in lysine concentration (λ_ex_ = 334 nm, slit: 2.5/5.0 nm).

**Figure 6:**
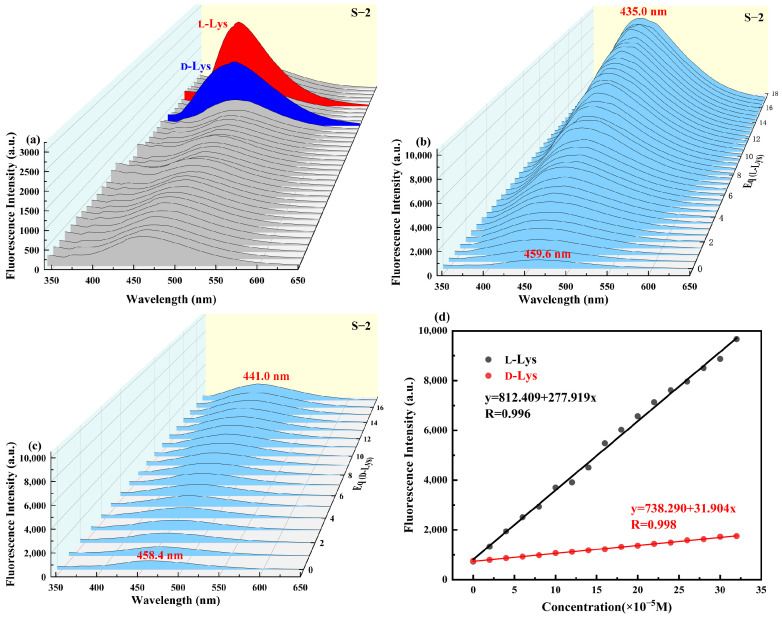
(**a**) Fluorescence emission spectra of S-2 (20 μM) with common amino acids (5 eq) in DMSO solvent; fluorescence spectra of S-2 (20 μM in DMSO, 1.0 eq) with (**b**) L-lysine or (**c**) D-lysine (in H_2_O, 0.5–17.0 eq); (**d**) fluorescence intensity of S-2 changed with increase in lysine concentration (λ_ex_ = 331 nm, slit: 5.0/5.0 nm).

The optical sensing behavior of S-1 and S-2 toward common amino acids in DMSO solution was investigated by fluorescence emission spectroscopy. S-1 and S-2 were dissolved in DMSO to form a test solution with a concentration of 20 μM. The 18 pairs of amino acids commonly used in the experiment were prepared into a solution with a concentration of 50 mM in ultra-pure water. At room temperature, each amino acid (5 eq) was added to the test solution in turn and thoroughly mixed for fluorescence spectrum testing. As shown in Figure 5a, in the DMSO solution with S-1, only L-lysine had a significant fluorescence response, and the fluorescence intensity increased from 783 to 1030. However, the fluorescence intensity did not change when D-lysine was added under the same conditions. The fluorescence intensity I_L_/I_D_ value was 1.3 (5 eq). The fluorescence shift occurred as a blue shift, indicating that the electrons on the L-lysine amino group were transferred to the electron-deficient probe seal. When L-lysine was added to the DMSO solution with S-2, the fluorescence intensity was significantly higher than that of D-lysine, and the I_L_/I_D_ value was 2.1 (5 eq), as shown in Figure 6a. Therefore, the two novel chiral fluorescence probes S-1 and S-2 have obvious chemical selectivity for lysine.

In order to further illustrate the enantioselectivity of lysine, the fluorescence titration experiments of S-1 and S-2 for lysine were studied. The fluorescence spectrum shown in Figure 5b demonstrates that 5–100 eq L/D-lysine was added to S-1 successively. As shown in Figure 5b, the fluorescence intensity of S-1 was enhanced with the increase in L-lysine concentration. Moreover, the emission wavelength of S-1 in DMSO was 506.8 nm, and the emission wavelength of the mixture was continuously blue-shifted with the increase in L-lysine concentration. When 100 eq L-lysine was added, the emission wavelength of the mixture was 444.6 nm and a blue shift of 66.2 nm occurred. However, as shown in Figure 5c, the fluorescence intensity of the system increased slowly with the increase in D-Lys concentration, accompanied by a slight blue shift of ~4.8 nm. As shown in Figures S13 and S14, the enantioselective fluorescence enhancement ratio [ef = (I_L_ − I_0_)/(I_D_ − I_0_); I_D_, I_L_, and I_0_: the strongest fluorescence intensity with and without D/L-Lys, respectively] was 10.0, showing high enantioselectivity. The excellent linear relationship between them is shown in Figure 5d.

L-lysine and D-lysine of 0.5–17 eq were added to the DMSO solution with S-2, and the fluorescence spectra were tested. As shown in Figure 6b,c, when L-lysine was added, the fluorescence intensity of the mixture was much higher than when D-lysine was added at the same concentration. When 17 eq L-lysine was added, the system had a blue shift of 24.6 nm, and when the same concentration of D-lysine was added, the system had a blue shift of 17.4 nm. As shown in Figure S2, the enantioselective fluorescence enhancement ratio was 10.3, showing high enantioselectivity. Meanwhile, the fluorescence showed an excellent linear relationship with the concentration (Figure 6d) upon the addition of L/D-lysine in different equivalent amounts (0.5–17 eq) to S-2.

**Figure 7:**
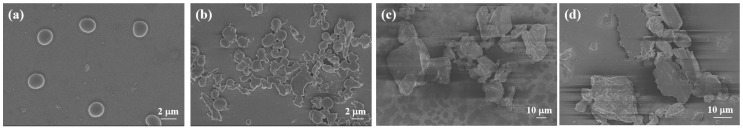

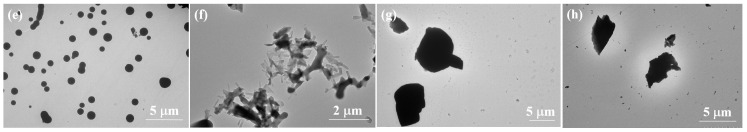
(**a**) The SEM image of S-1 with L-Lys in EtOH. (**b**) The SEM image of S-1 with D-Lys in EtOH. (**c**) The SEM image of S-2 with L-Lys in EtOH. (**d**) The SEM image of S-2 with D-Lys in EtOH. (**e**) The TEM image of S-1 with L-Lys in EtOH. (**f**) The TEM image of S-1 with D-Lys in EtOH. (**g**) The TEM image of S-2 with L-Lys in EtOH. (**h**) The TEM image of S-2 with D-Lys in EtOH.

To further characterize the morphological changes of the compounds before and after binding to lysine, we also used SEM and TEM. As shown in Figure 7a,b,e,f, when L-lysine was added to S-1, the self-assembled vesicle structure of S-1 itself became more regularly spherical and evenly dispersed. However, the addition of D-lysine did not change much, and the mostly aggregated large vesicle structure remained. Again, as shown in Figure 7c,d,g,h, when D/L lysine was added to S-2, the morphology of the masses in S-2 hardly changed significantly at all. This suggests that unlike S-1, S-2 does not cause changes in its own morphology when recognizing D/L-lysine. Therefore, *S*-1 can also recognize D/L-lysine through morphological changes in self-assembly.

**Figure 8:**
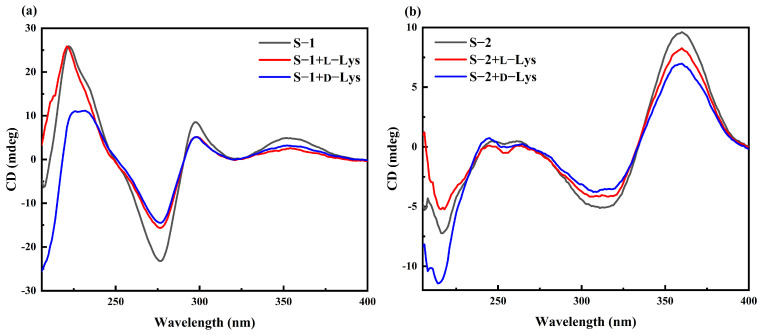
(**a**) The CD spectrum shows changes in S-1, S-1 with L-Lys, and S-1 with D-Lys. (**b**) The CD spectrum shows changes in S-2, S-2 with L-Lys, and S-2 with D-Lys.

Adding amino acids of different configurations to chiral fluorescent probes might also have an effect on their CD signal. Next, we performed CD spectroscopy on the probes S-1 and S-2. Figure 8a,b show that there was little apparent effect on the CD signals of S-1 and S-2 when D/L-Lys was added. The results suggest that these two probes may not be able to effectively recognize different conformations of lysine by circular dichroism.

### 2.5. DFT Calculations

**Figure 9:**
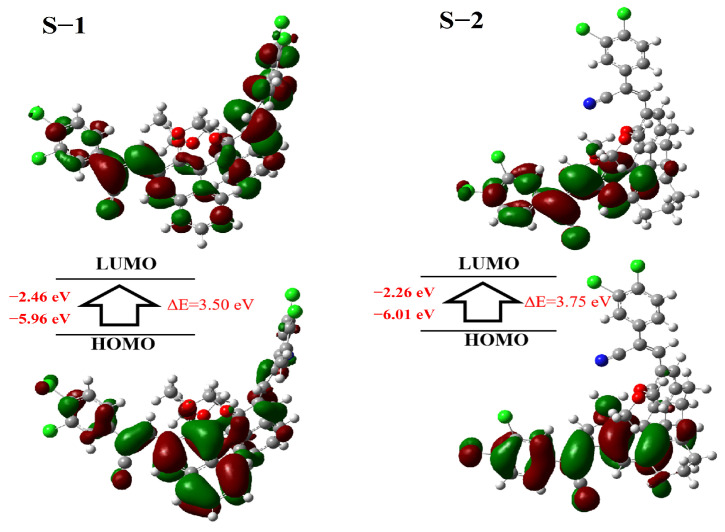
The HOMO/LUMO orbitals of S-1 and S-2 by DFT using Gaussian 16.

In order to analyze the electronic properties and geometric structures of the two molecules, we were further simulated and optimized at the B3LYP/6-31G(d) level. The HOMO and LUMO orbital distributions of S-1 and S-2 are shown in Figure 9. Their HOMO and LUMO levels and Eg are −5.96, −2.46, and 3.50 eV, and −6.01, −2.26, and 3.75 eV, respectively.

The study of different conformations of lysine revealed that L-Lys significantly enhanced the fluorescence intensity. This may be due to the higher electron-donating ability of the lone-pair electrons of the N atom in the amino group at the terminal position of L-Lys, which enhances the ability of the electron-withdrawing groups on both materials. The electron transfer capacity within the structure of the D-A-D molecule is improved, causing an intramolecular charge transfer effect (ICT). Also, due to the specific chiral configuration of L-lysine, the steric hindrance is small, so the two materials have strong fluorescence-specific recognition of L-lysine [27,28,29].

## 3. Materials and Methods

All experimental compounds and solvents were purchased externally. SEM images were taken using the Philips-FEI Tecnai G2S-Twin microscope (FEI, Hillsboro, OR, USA). The measurement of optical rotation data was carried out on the Rudolph AUTOPOL IV automatic polarimeter (Hackettstown, NJ, USA). CD spectra data were detected by using a JASCO810 spectrometer (Tokyo, Japan). Electrospray mass spectra were acquired using the Bruker Amazon SL ion trap mass (Karlsruhe, Germany). The NMR spectra mentioned in this paper were all from the Bruker AM-400WB spectrometer (Karlsruhe, Germany), and the melting point was observed by using an X-4 melting point measuring instrument (Beijing Tech Instrument, Beijing, China). Infrared data were obtained from measurements with a Bruker VERTEX 70 Fourier Transform Infrared Spectrometer (Karlsruhe, Germany). The fluorescence spectral data were measured on a Hitachi F-7100 fluorescence spectrophotometer (Hitachi High-Technologies, Tokyo, Japan), and the UV-Vis spectra were measured on a PerkinElmer Lambda-900 UV-Vis spectrophotometer (Hopkinton, MA, USA).

### 3.1. Synthesis of Probe S-1

To compound 3 (200.0 mg, 0.46 mmol) in ethanol (10 mL), sodium methylate (75.4 mg, 1.39 mmol) and 3,4-dichlorophenylacetonitrile (190.3 mg, 1.02 mmol) were added. The reaction mixture was stirred at room temperature for 10 h, and the color of the reaction system was orange. After TLC monitoring, the raw material disappeared, and then, the reaction mixture was combined with CH_2_Cl_2_ (3 × 20 mL) and H_2_O (20 mL) for extraction. The extracted organic phase was washed with brine (20 mL), dried with anhydrous Mg_2_SO_4_, and then concentrated under reduced pressure to remove the solvent. The crude product was purified by column chromatography on silica gel (PE/EA = 20/1, *v*/*v*) to obtain a yellow solid with a yield of 69% (246.0 mg). ${\left[ɑ\right]}_{D}^{25}$ 206 (c = 1, CH_3_CN). m.p. 62–67 °C. HR-MS (ESI+): calcd for C_42_H_28_C_l4_N_2_O_4_ [M + Na] + 787.0701, found 787.0707. ^1^H NMR (400 MHz, Chloroform-*d*) *δ* 8.86 (s, 2H), 8.21 (s, 2H), 8.06 (d, *J* = 8.2 Hz, 2H), 7.86 (d, *J* = 2.0 Hz, 2H), 7.60 (dd, *J* = 8.5, 2.1 Hz, 2H), 7.57–7.50 (m, 4H), 7.41 (t, *J* = 7.7 Hz, 2H), 7.27 (s, 1H), 7.25 (s, 1H), 4.62–4.55 (m, 4H), 2.84 (s, 6H). ^13^C NMR (101 MHz, Chloroform-*d*) *δ* 152.02, 139.19, 134.65, 134.12, 133.42, 130.88, 130.26, 129.85, 129.73, 129.11, 128.43, 127.56, 127.05, 126.03, 125.76, 125.34, 124.97, 116.99, 110.76, 100.04, 56.90.

### 3.2. Synthesis of Probe S-2

3,4-dichlorophenylacetonitrile (280 mg, 1.50 mmol) and sodium methanol (111 mg, 2.05 mmol) were added to 10 mL ethanol solution of compound 5 (300 mg, 0.68 mmol). The reaction system was stirred at room temperature for 10 h, and the disappearance of the raw materials was monitored by TLC. Then, the reaction mixture was extracted with CH_2_Cl_2_ (20 mL × 3) and H_2_O (20 mL). The resulting organic phase was washed with brine (20 mL), dried with anhydrous Mg_2_SO_4_ for 20 min, and concentrated under reduced pressure to remove the solvent. The crude product was purified by silica gel column chromatography (PE/EA = 40/1, *v*/*v*), and the yield of yellow solid was 61% (321 mg). ${\left[ɑ\right]}_{D}^{25}$ 208 (c = 1, CH_3_CN). m.p. 134–139 °C. HR-MS (ESI+): calcd for C_42_H_36_C_l4_N_2_O_4_ [M + Na] + 795.1327, found 795.1320. ^1^H NMR (400 MHz, Chloroform-*d*) *δ* 7.98 (s, 3H), 7.94 (s, 1H), 7.79 (s, 2H), 7.51 (s, 4H), 4.69 (s, 4H), 3.02 (s, 6H), 3.00–2.84 (m, 4H), 2.54–2.40 (m, 2H), 2.40–2.26 (m, 2H), 1.92–1.79 (m, 4H), 1.79–1.69 (m, 4H). ^13^C NMR (101 MHz, Chloroform-*d*) *δ* 152.74, 141.82, 139.53, 134.78, 134.66, 133.63, 133.31, 131.10, 128.64, 127.72, 125.16, 124.81, 117.50, 109.18, 100.27, 57.05, 29.66, 28.43, 22.76.

All synthetic schemes, NMR, MS and IR profiles can be viewed in the Supplementary Materials (Schemes S1–S4 and Figures S1–S12).

### 3.3. Preparation of Fluorescent Probe Solutions

Probes S-1 and S-2 were dissolved in chromatography-pure THF or DMSO solvents at a concentration of 20 μM. Eighteen pairs of common amino acids were dissolved in water and configured into a solution with a concentration of 0.05 M.

## 4. Conclusions

In summary, we have achieved the successful synthesis of two AIE-active compounds S-1 and S-2 via a combination of chiral BINOL and H_8_-BINOL aldehyde derivatives together with an achiral 3,4-dichlorophenylacetonitrile utilizing the well-established Knoevenagel reaction. These two unique compounds display exceptional AIE properties, as illustrated through their fluorescence, ultraviolet, and circular dichroism (CD) spectra. We examined the photophysical trends of S-1 and S-2 through UV-Vis absorption and fluorescence emission spectral analysis. Notably, the UV-Vis spectrum of S-1 in tetrahydrofuran (THF) solution highlights the characteristic absorbance band of BINOL at 290 nm, while the absorption peak at 334 nm mirrors the intramolecular charge transfer (ICT) from the BINOL electron donor to the electron-accepting (1-cyano-1-(3,4-dichlorophenyl)) methylene group. Concerning their fluorescence enhancements, we observed enantioselective values of 10.0 and 10.3 for S-1 and S-2 toward lysine, respectively, along with a distinct blue shift during the recognition event. This observation is further supported by changes in their microstructure, detailed molecular architecture, and CD signal. It is noteworthy that our study significantly broadens the scope of designing small organic fluorescent molecules with AIE activity. Moreover, it presents a novel approach for enantioselective recognition of lysine.

## Data Availability

The original contributions presented in the study are included in the article/Supplementary Materials, further inquiries can be directed to the corresponding authors.

## Supplementary Materials

The following supporting information can be downloaded at: https://www.mdpi.com/article/10.3390/ijms251910666/s1.
