# Supplementary material for: Novel Chiral Self-Assembled Nano-Fluorescence Materials with AIE Characteristics for Specific Enantioselective Recognition of L-Lysine
Source: Int J Mol Sci. 2024 Oct 3;25(19):10666. doi: 10.3390/ijms251910666 (PMC11476731; doi:10.3390/ijms251910666)
Supplement: Supplementary file 1 [file ijms-25-10666-s001.zip › ijms-3214019-supplementary.pdf]

Supporting Information

Two Novel Chiral nano-Fluorescence Materials Show Distinctive Enantiospecific Recognition towards L-Lysine

Contents

1. Synthesis and characterization of compounds 2
 1.1. Characterization of S-1 2
 1.2. Synthesis and characterization of compound 4 4
 1.3. Synthesis and characterization of the compound 5 6
 1.4. Characterization of S-2 8
 1.5 Synthesis and characterization of R-2 10
2. Fluorescence spectra of S-1 and S-2 with lysine 11
 2.1 Fluorescence spectra of S-1 with lysine 11
 2.2 Fluorescence spectra of S-2 with lysine 11
 2.3 CPL spectra of S-1and S-2 12
 2.4 CD spectra of R/S-2 12

1. Synthesis and characterization of compounds

1.1. Characterization of S-1

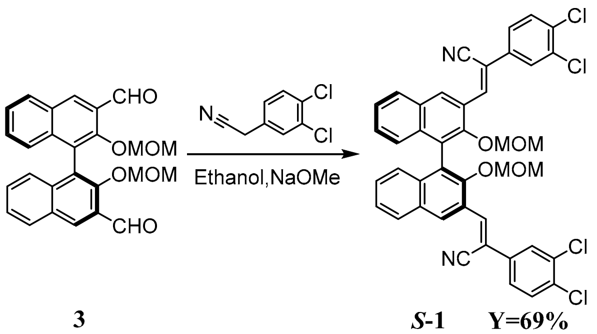

3 ***S*-1** **Y=69%**

Scheme S1. Synthesis of S-1.

^{1}H NMR spectra of S-1

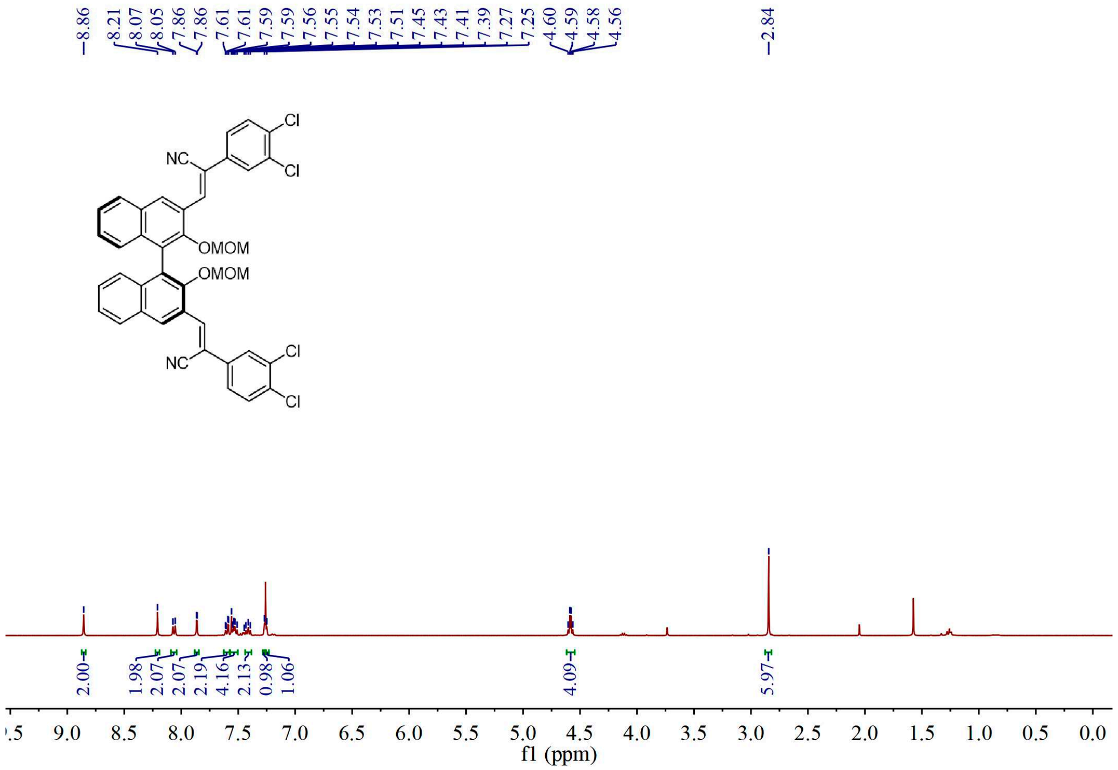

Figure S1. ^{1}H NMR of S-1.

^{13}C NMR spectra of S-1

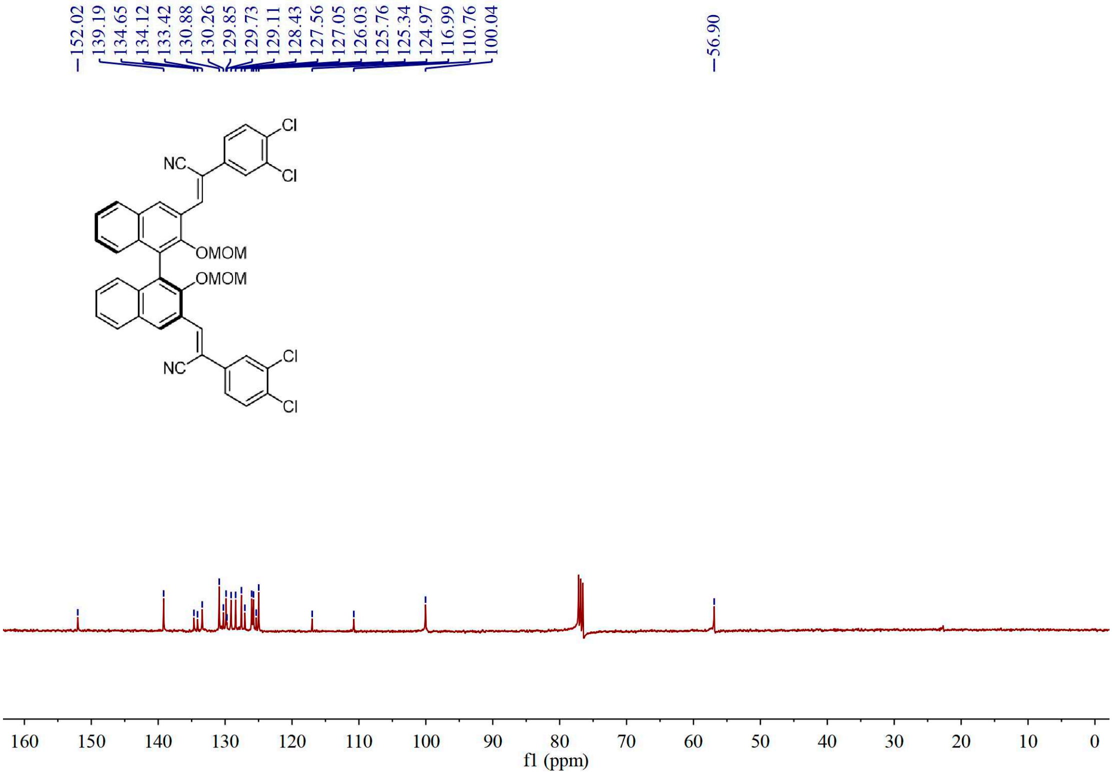

Figure S2. ^{13}C NMR of S-1.

Infrared spectrogram of S-1

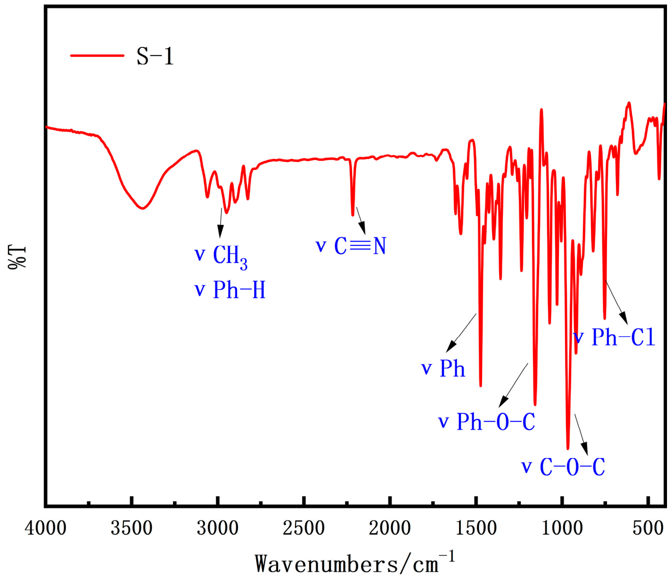

Figure S3. Infrared spectrogram of S-1.

HRMS of S-1

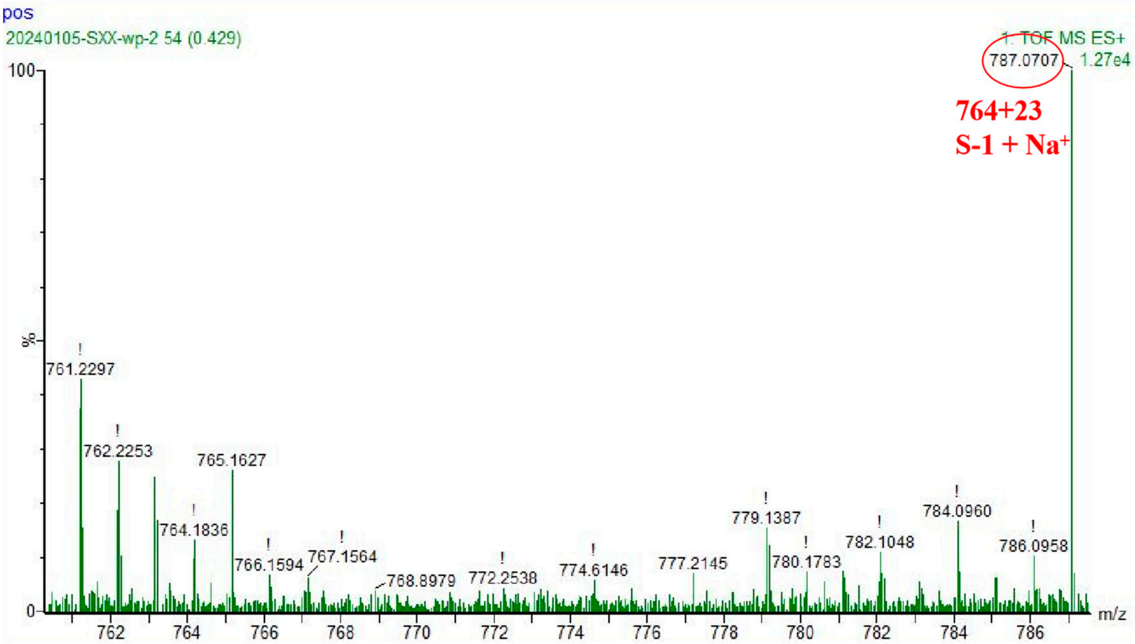

Figure S4. HRMS of S-1.

1.2. Synthesis and characterization of compound 4

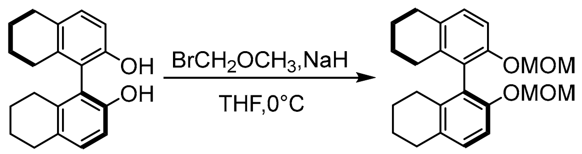

***S*-H$_8$-BINOL** **4** **Y=91%**

Scheme S2. Synthesis of compound **4.**

(S)-H$_8$-BINOL (3.00 g, 10.19 mmol) dissolved in tetrahydrofuran (15 ml) was added dropwise to a solution of sodium hydride (1.20 g, 50.95 mmol) in tetrahydrofuran (5 ml) under argon protection. The reaction system was stirred at 0 °C for 30 min and then bromomethyl methyl ether (3.6 ml, 30.57 mmol) was added dropwise to the reaction system. At this point bubbles were observed and the colour of the mixture changed from grey to white turbid liquid, which was brought to room temperature and stirring was continued for 10 h. After the disappearance of raw material points was monitored by TLC, the excess sodium hydride in the reaction system was quenched with H_2O (20 ml), and the mixture was extracted with EA (10 ml×3). The extracted organic phase was washed in brine (20 ml), dried with anhydrous Mg_2SO_4, and then concentrated to remove solvent under reduced pressure. The crude product was separated and purified by column chromatography (PE/EA=35/1, v/v) on silica gel to obtain a green viscous solid (3.54 g) with a yield of 91%. ^{1}H NMR (400 MHz, Chloroform-*d*) δ 7.15-6.97 (m, 2H), 5.11-4.94 (m, 2H), 3.32 (s, 3H), 2.81 (t, *J* = 6.2 Hz, 2H), 2.45-2.08 (m, 2H), 1.83-1.64 (m, 4H). ^{13}C NMR (101 MHz, Chloroform-*d*) δ 152.25, 136.79, 130.93, 128.84, 127.14, 112.78, 94.81, 55.60, 29.47, 27.31, 23.25, 23.14.

^{1}H NMR spectra of compound **4**

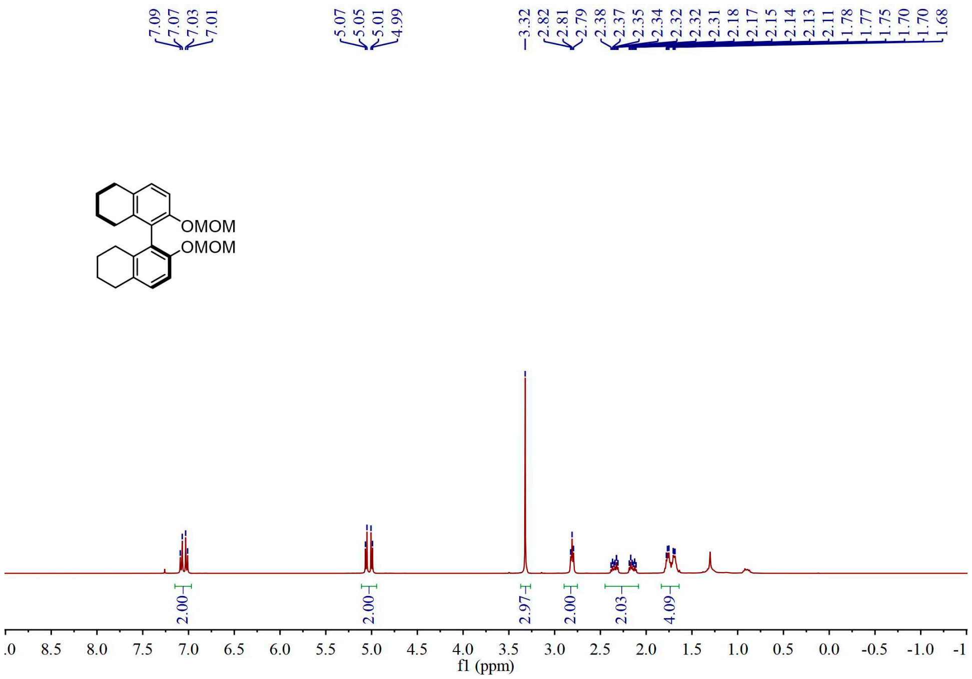

Figure S5. ^{1}H NMR of compound **4**.

^{13}C NMR spectra of compound **4**

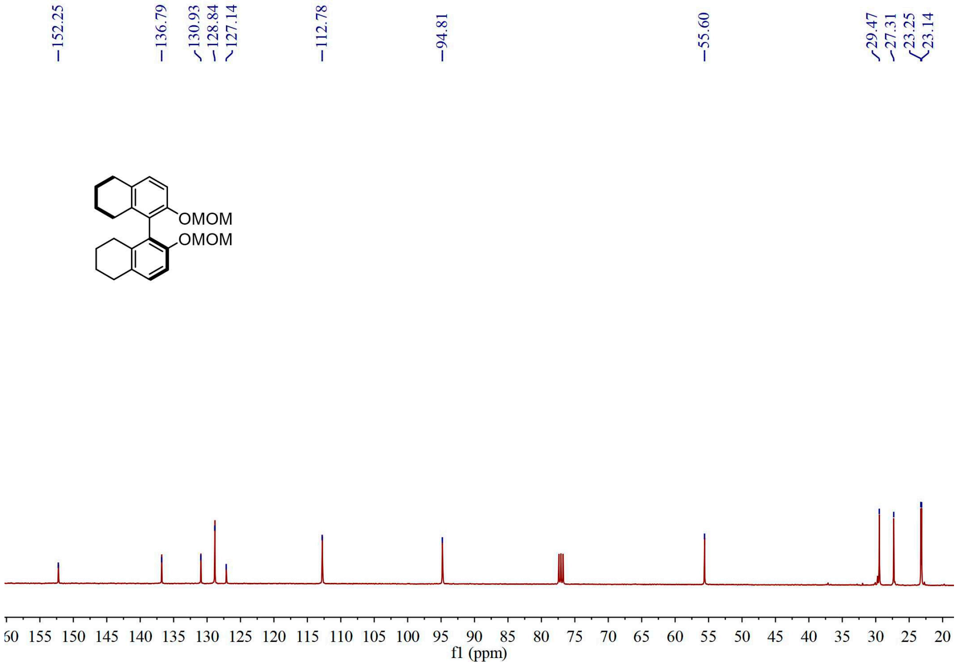

Figure S6. ^{13}C NMR of compound **4**.

1.3. Synthesis and characterization of the compound 5

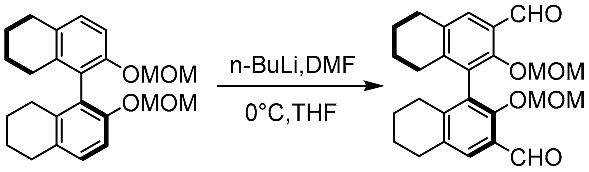

4 5 Y=72%

Scheme S3. Synthesis of compound **5.**

Under the protection of argon, n-butyl lithium (2.5 M, 27.78 mmol, 11.11 ml) was added to the solution of compound **4** (3.70 g, 9.26 mmol) in the THF drop by drop, and the color of the reaction system changed from transparent to dark brown after stirring at 0 ° C for 1h. Then N, N-dimethylformamide (2.14 ml, 27.78 mmol) was slowly added to the mixture. After rising to room temperature, the mixture was stirred for another 2 h. The color of the reaction system changed from dark brown to milky white. A saturated ammonium chloride solution was added to neutralize the excess n-butyl lithium, and the residue was extracted with EA (20 ml×3). After the organic phase was washed with brine (20 ml), dried with anhydrous Mg_2SO_4 for 20 min, then concentrated to remove solvent under reduced pressure. The crude product was separated and purified by column chromatography (PE/EA=10/1, v/v) on silica gel to obtain a yellow solid (3.54 g) with a yield of 72%. ^{1}H NMR (400 MHz, Chloroform-*d*) δ 10.24 (s, 2H), 7.63 (s, 2H), 4.87-4.77 (m, 4H), 2.99 (s, 6H), 2.84 (t, *J* = 6.1 Hz, 4H), 2.45 (s, 2H), 2.25 (s, 2H), 1.77 (d, *J* = 6.5 Hz, 4H), 1.69 (d, *J* = 6.0 Hz, 4H). ^{13}C NMR (101 MHz, Chloroform-*d*) δ 190.16, 155.47, 145.22, 134.30, 130.84, 126.97, 100.54, 56.61, 29.20, 28.26, 22.36.

^{1}H NMR spectra of the compound **5**

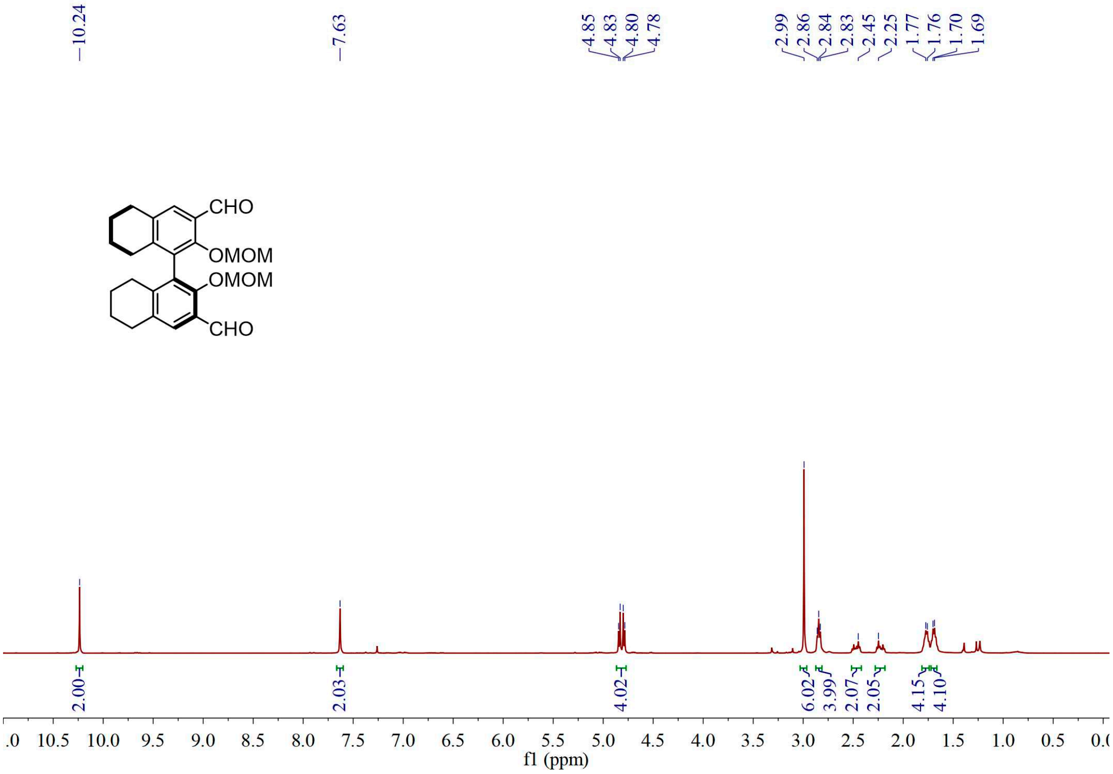

Figure S7. ^{1}H NMR of compound **5**.

^{13}C NMR spectra of the compound **5**

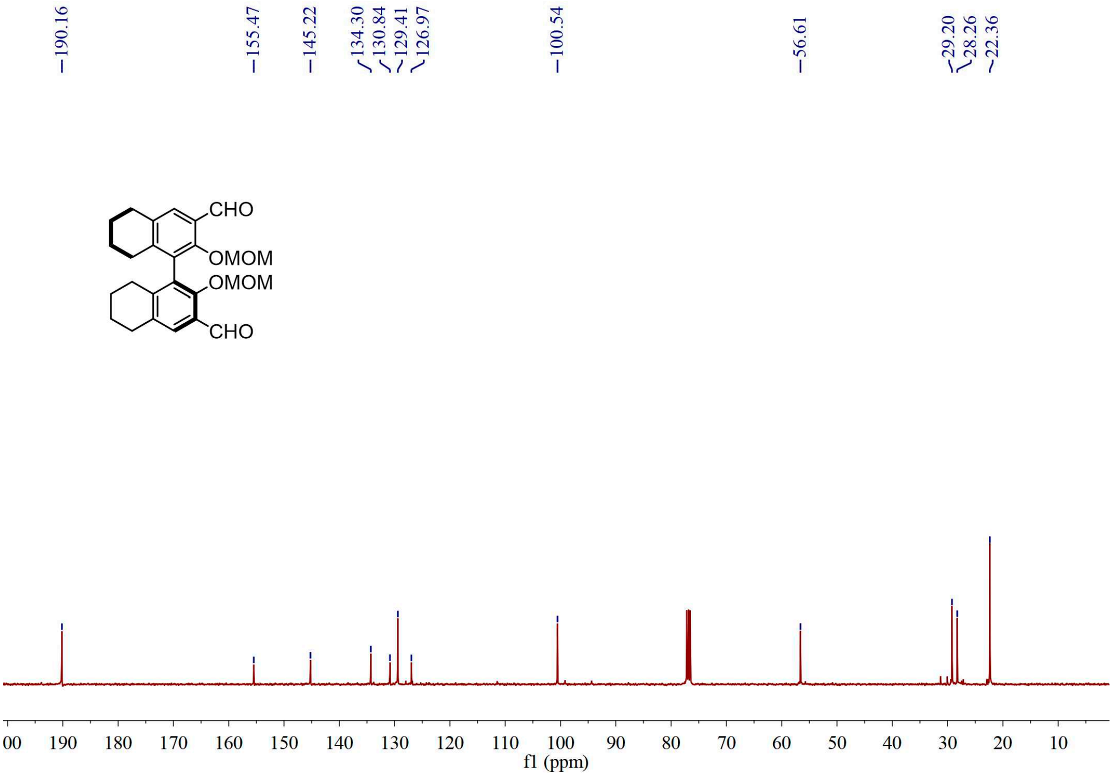

Figure S8. ^{13}C NMR of compound **5**.

1.4. Characterization of S-2

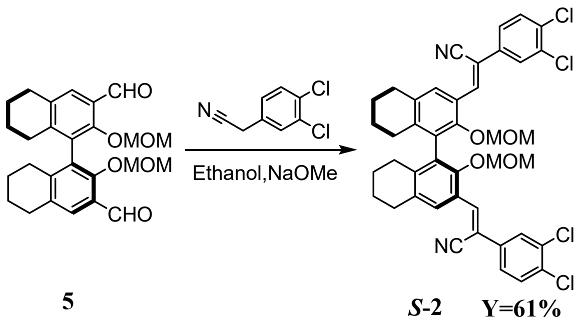

Scheme S4. Synthesis of S-**2.**

^{1}H NMR spectra of S-**2**

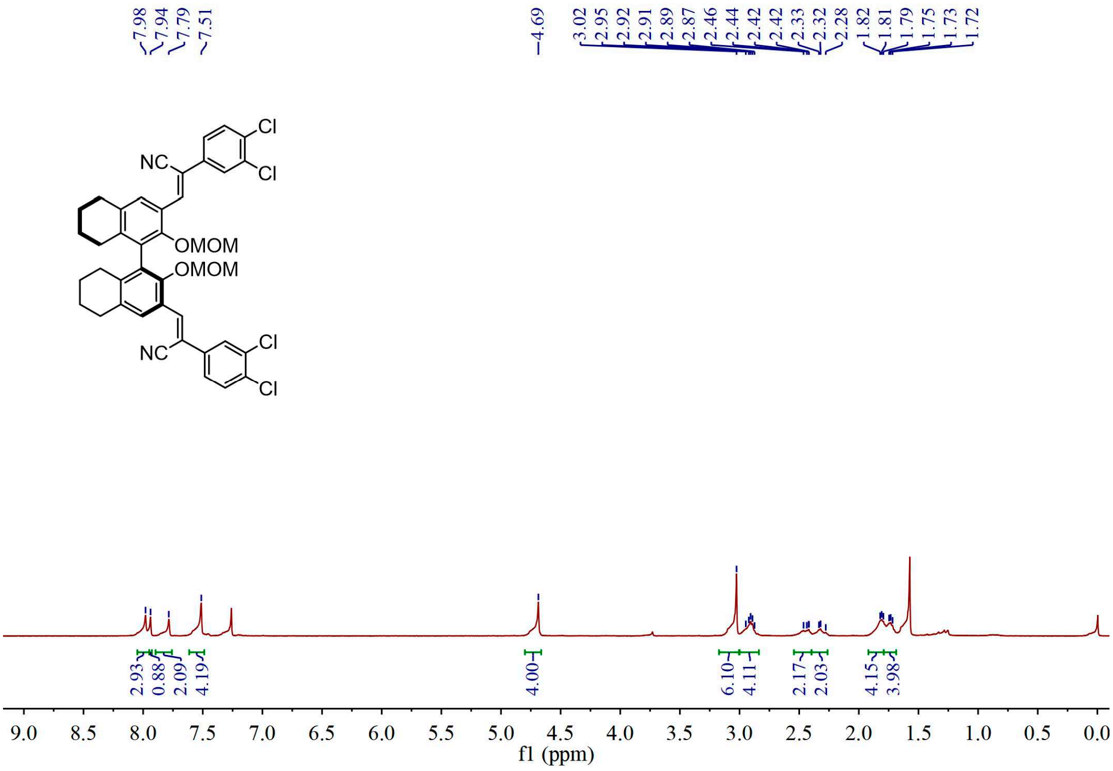

Figure S9. ^{1}H NMR of S-2.

^{13}C NMR spectra of S-**2**

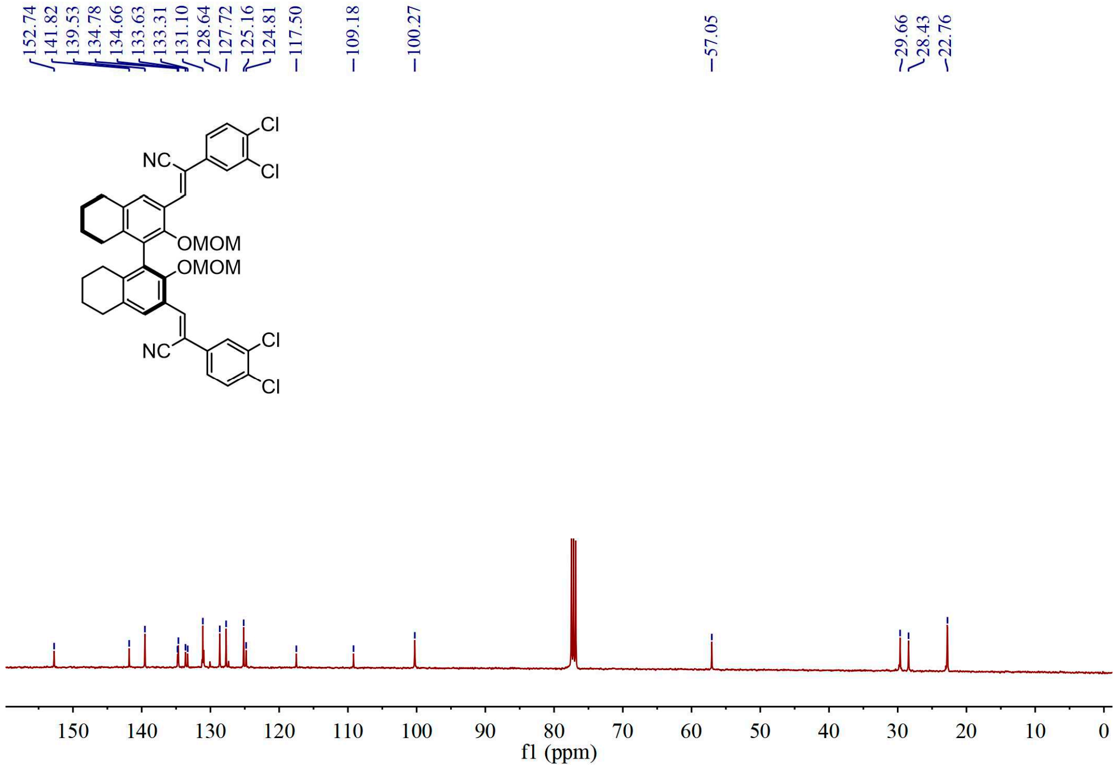

Figure S10. ^{13}C NMR of S-2.

Infrared spectrogram of S-2

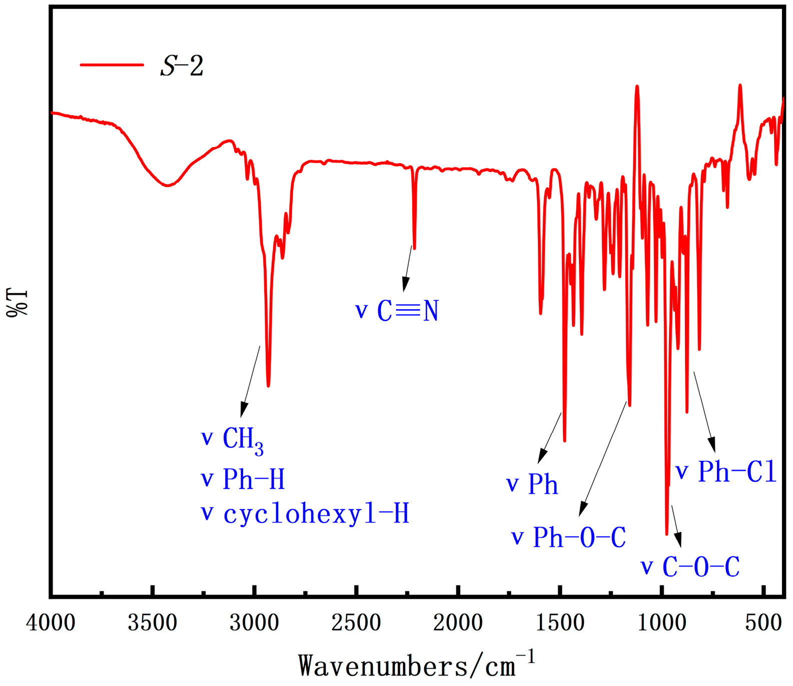

Figure S11. Infrared spectrogram of S-2.

HRMS of S-2

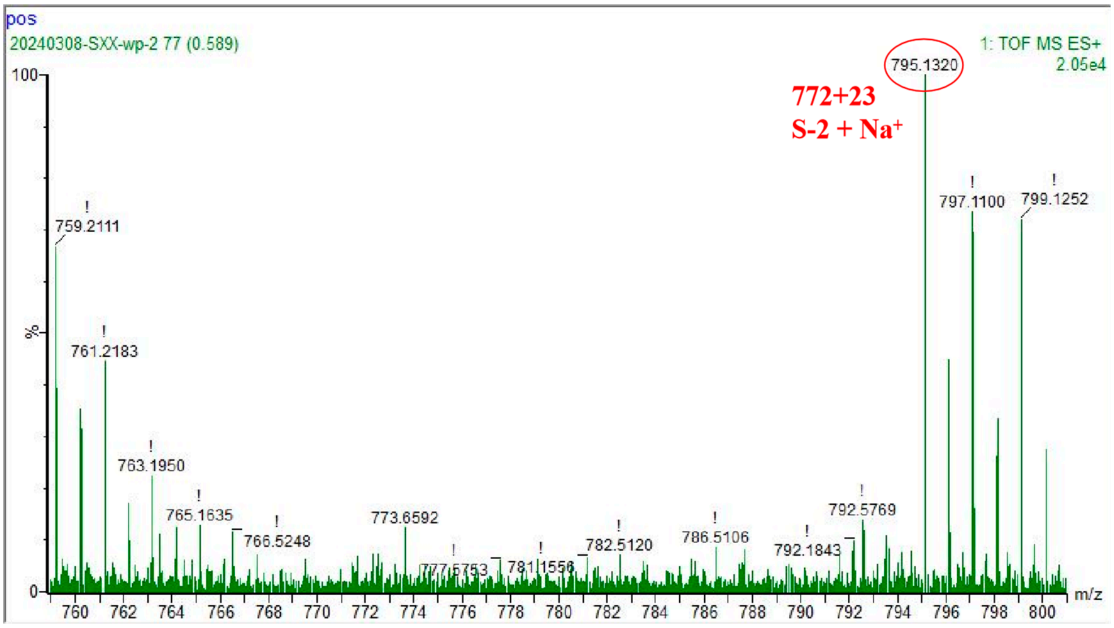

Figure S12. HRMS of S-2

1.5 Synthesis and characterization of R-2

Compound 5(R) (300 mg, 0.68 mmol) and sodium methanol (111 mg, 2.05 mmol) were accurately weighed in a 50 mL round-bottomed flask, 10 mL of ethanol was added and stirred until dissolved, followed by the addition of 3,4-dichlorophenylacetonitrile (280 mg, 1.50 mmol) dissolved in a small amount of ethanol, and the reaction was continued at room temperature for 10 h. TLC monitoring (PE:EA=6:1, R_f=0.46), the raw material disappeared, added water and extracted with CH_2Cl_2 (10 mL×3), the organic phases obtained from the three extractions were combined, washed with saturated NaCl solution, then dried with anhydrous Mg_2SO_4 for 20-30 min, filtered and distilled under reduced pressure to remove the solvent, and then separated and purified by column chromatography (PE:EA=38:1). Yellow solid R-2 290 mg in 55% yield. R-2 $[\alpha]_D^{25}$ -208 (c=1, CH_3CN). ^1H NMR (400 MHz, Chloroform-*d*) δ 7.98 (s, 3H), 7.94 (s, 1H), 7.78 (s, 2H), 7.51 (s, 4H), 4.69 (s, 4H), 3.02 (s, 6H), 2.97 - 2.82 (m, 4H). 2.82 (m, 4H), 2.50 - 2.38 (m, 2H), 2.37 - 2.26 (m, 2H), 1.85 - 1.78 (m, 4H), 1.77 - 1.66 (m, 4H).

2. Fluorescence spectra of S-1 and S-2 with lysine

2.1 Fluorescence spectra of S-1 with lysine

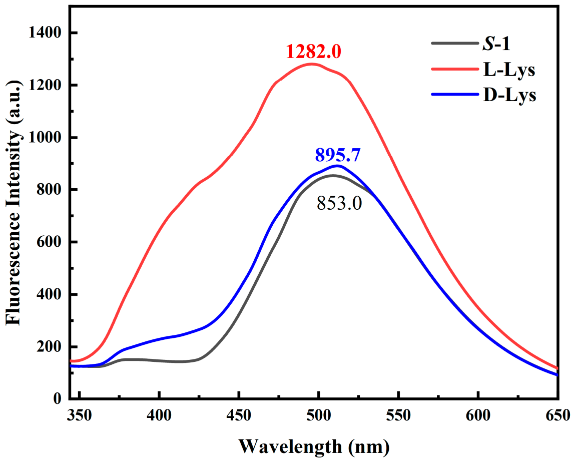

Figure S13. Fluorescence spectra of S-1 with lysine (10.0 eq) in DMSO solutions.

2.2 Fluorescence spectra of S-2 with lysine

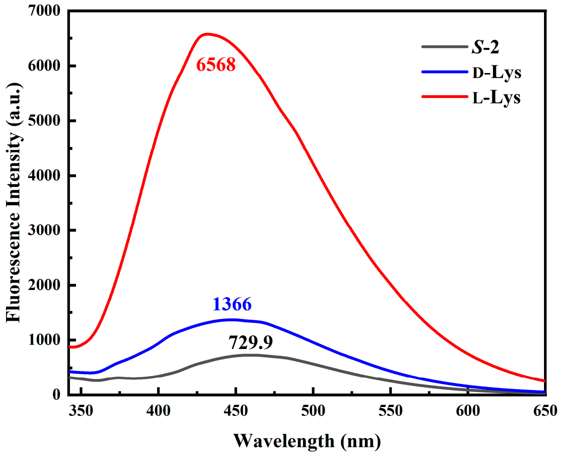

Figure S14. Fluorescence spectra of S-2 with lysine (10.0 eq) in DMSO solutions.

2.3 CPL spectra of S-1and S-2

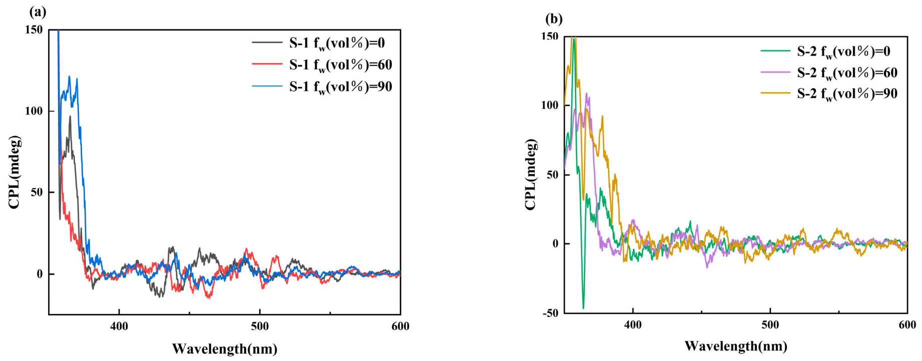

Figure S15. CPL spectra of S-1 and S-2.

2.4 CD spectra of R/S-2

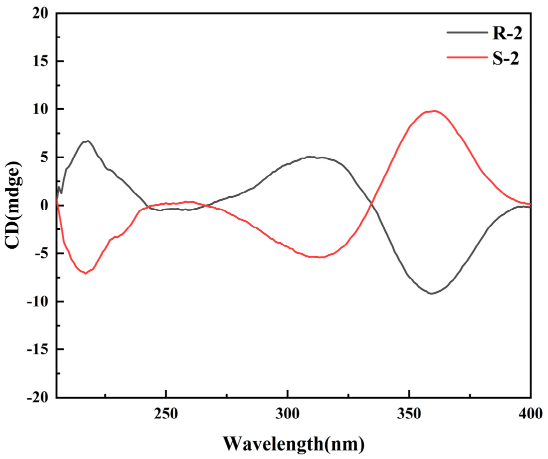

Figure S16. CD spectra of R/S-2.
